# Endoscopic submucosal dissection for papillary early gastric carcinoma: Insights from a large-scale analysis of post-gastrectomy pathology specimens

**DOI:** 10.1097/MD.0000000000032085

**Published:** 2022-12-16

**Authors:** Jung Hwan Lee, Ju Yeon Oh, Young-Il Kim, Jong Yeul Lee, Chan Gyoo Kim, Il Ju Choi, Keun Won Ryu, Young-Woo Kim, Soo-Jeong Cho

**Affiliations:** a Center for Gastric Cancer, National Cancer Center, Goyang, Korea; b Department of Hospital Medicine, Inha University Hospital, Inha University School of Medicine, Incheon, Korea; c Division of Gastroenterology, Department of Internal Medicine and Liver Research Institute, Seoul National University, Seoul, Korea.

**Keywords:** adenocarcinoma, early gastric cancer, endoscopic mucosal resection, lymph node metastasis, papillary

## Abstract

Gastric papillary adenocarcinoma is considered a differentiated adenocarcinoma in the current endoscopic submucosal dissection indication guidelines. However, the safety of endoscopic submucosal dissection remains controversial. Currently, data regarding which papillary early gastric cancer should be considered for endoscopic submucosal dissection are unavailable. Thus, the aim of this study was to investigate lymph node metastasis and the safety of endoscopic submucosal dissection in patients with papillary early gastric cancer. This observational study recruited 4264 consecutive patients with early gastric cancer who underwent curative gastrectomy between October 2000 and December 2017 at the National Cancer Center, Korea. Of these, 45 had pathologically confirmed papillary early gastric cancer, 2106 had differentiated non-papillary early gastric cancer, and 2113 had undifferentiated early gastric cancer. Logistic regression analysis was performed to identify risk factors for lymph node metastasis. Mucosal tumors were less common in papillary early gastric cancer (37.9%) than in differentiated non-papillary early gastric cancer (48.8%) and undifferentiated early gastric cancer (60.4%) (both *P *< .001). Lymph node metastasis was more common in papillary early gastric cancer (20.0%) than in differentiated non-papillary early gastric cancer (9.2%) and undifferentiated early gastric cancer (11.7%; both *P *< .001). In multivariate analysis, non-mixed-type papillary early gastric cancer showed marginally increased odds of lymph node metastasis than differentiated early gastric cancer (odds ratio [OR]: 2.5, 95% confidence interval [CI]: 1.0–6.3). Rates of lymph node metastasis (1/10, 10%) and angiolymphatic invasion (2/10, 20%) for papillary early gastric cancer meeting expanded criteria were higher than those for other histology types meeting endoscopic submucosal dissection absolute or expanded criteria (*P* = .03 and *P* < .001, respectively). Endoscopic submucosal dissection should be considered carefully for papillary early gastric cancer, especially if it meets expanded endoscopic submucosal dissection indications since it is associated with high rates of submucosal invasion and lymph node metastasis.

## 1. Introduction

Endoscopic submucosal dissection (ESD) is a standard treatment for limited-stage early gastric cancer (EGC).^[1]^ It has benefits of being minimally invasive, yielding higher rates of both en bloc and complete resection than endoscopic mucosal resection.^[2]^ However, it is important to evaluate the risk of lymph node metastasis of EGC before performing an ESD. Indications for ESD have been based on reports that certain groups of patients with EGC have a minimal risk or no likelihood of developing lymph node metastasis, thereby shifting the risk-benefit ratio in favor of ESD.^[3]^ Current indication criteria include differentiated tumors with minimal submucosal invasion or ulceration, although some oncology societies have expanded their criteria to include undifferentiated EGCs without submucosal invasion and differentiated tumors with minimal invasion or with ulcers but no invasion.^[4–6]^

Papillary gastric carcinoma is a rare histologic entity among gastric adenocarcinomas. It is classified as a differentiated tumor according to ESD guidelines.^[5,7]^ Clinicopathologic characteristics of papillary EGC (P-EGC) include a low overall survival rate and high rates of lymph node metastasis and submucosal invasion.^[8–10]^ Recent studies have reported that ESD for P-EGC should be considered carefully because of a high rate of lymph node metastasis, especially when expanded criteria for ESD are applied.^[11,12]^ This leads to the question of whether it is safe to apply the same ESD indication criteria for tubular gastric adenocarcinomas and P-EGC. Data regarding which P-EGCs should be considered for ESD are currently unavailable.

Thus, the objectives of this study were to determine clinicopathologic characteristics and lymph node metastasis rates in patients with P-EGC and to investigate the safety of current indications for ESD in these patients based on the hypothesis that P-EGC might have more lymph node metastasis rates.

## 2. Materials and Methods

### 2.1. Patients

**Figure 1.**
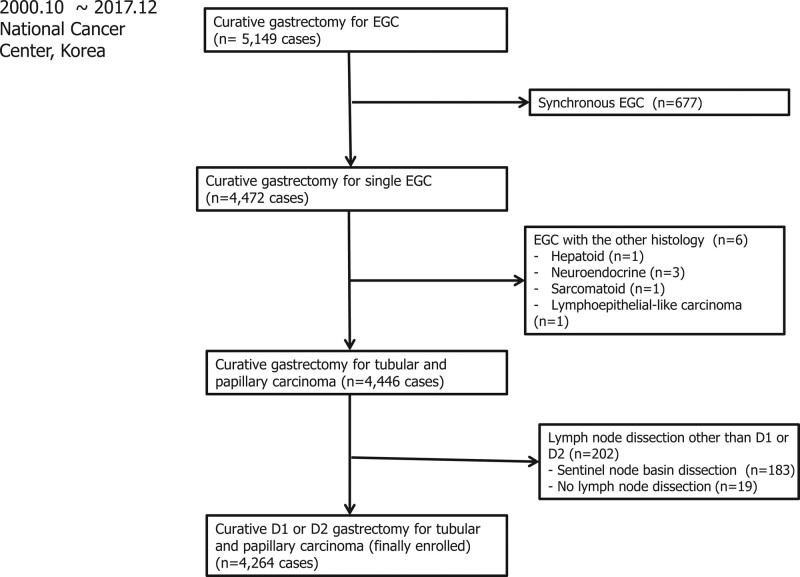
Selection of patients with early gastric cancer (EGC).

A total of 5149 consecutive patients with EGC underwent curative gastrectomy between October 2000 and December 2017 in the Center for Gastric Cancer at the National Cancer Center, Korea. In this retrospective cross-sectional study, patients who underwent curative gastrectomy with D1 + or D2 lymphadenectomy for primary EGC were included.^[5]^ A total of 677 patients with synchronous EGCs and 6 patients with malignancy other than adenocarcinoma were excluded. Of the remaining 4446 patients, 202 were excluded because the surgery did not involve D1 + or D2 lymphadenectomy (183 who underwent sentinel node basin dissection and 19 who did not undergo lymph node dissection).^[13]^ Thus, 4264 patients were enrolled in this study (Fig. 1). These patients were divided into 3 groups: differentiated EGC without P-EGC group, undifferentiated EGC group, and P-EGC group. This study was reviewed and approved by the ethics committee of the National Cancer Center (approval No. NCC 2020-0057). Patients were not required to give informed consent because the retrospective analysis used anonymous clinical data obtained after each patient agreed to undergo treatment with written consent.

### 2.2. Histologic classification

EGC morphologies were divided into 3 groups based on the Paris classification:^[14]^ Elevated lesions included protruded (0–I) and superficial elevated (0–IIa) types. Flat lesions included only superficial flat type (0–IIb). Depressed lesions included superficially depressed (0–IIc) and excavated type (0–IIc). The depth of tumor invasion into the submucosa was measured directly from the muscularis mucosae using a micrometer.^[15]^ Invasion < 500 µm was classified as sm1 according to endoscopic mucosal resection or ESD standards and invasion ≥ 500 µm was defined as sm2. Tumors were classified histologically into differentiated or undifferentiated types.^[5]^ The former included papillary adenocarcinoma and well-differentiated or moderately differentiated adenocarcinoma. The latter included poorly differentiated adenocarcinoma, signet-ring cell carcinoma, and mucinous carcinoma. Papillary adenocarcinomas were defined as tumors in which > 50% of the tumor area contained papillary structures composed of epithelial projections with a central fibrovascular core as a scaffold.^[7]^ They were subclassified as pure papillary adenocarcinoma, papillary adenocarcinoma mixed with other differentiated carcinomas, and papillary adenocarcinoma mixed with undifferentiated carcinoma.^[11]^

### 2.3. ESD indication criteria

Absolute indication criteria for ESD were: lesions limited to the mucosal layer, well and/or moderately differentiated adenocarcinoma, tumor ≤ 2 cm in diameter, and absence of ulcer or ulcer-scar tissue.^[4]^ Expanded indications were: well or moderately differentiated adenocarcinoma in the mucosal layer without an ulcer regardless of size; well or moderately differentiated adenocarcinoma <3 cm in diameter, which was ulcerated but still restricted to the mucosal layer; small (<2 cm) intramucosal cancer with undifferentiated histology; and well or moderately differentiated adenocarcinoma (with minimal submucosal invasion (≤500 µm [sm1]) and ≤3 cm in diameter.

### 2.4. Statistical analyses

Baseline characteristics are described according to the histologic group. Continuous variables are summarized as mean ± standard deviation. They were analyzed using one-way analysis of variance. For categorical variables, frequency and percentage were determined. Differences in distributions were estimated using Pearson’s chi-square test or Fisher’s exact test. To investigate risk factors for lymph node metastasis, odds ratios (ORs) and 95% confidence intervals (CIs) were calculated using logistic regression analysis. All statistical analyses were performed using the STATA software version 14.0 (STATA Corp., College Station, TX).

## 3. Results

Baseline characteristics of the 3 histologic groups are presented in Table 1. Of 4264 patients included in the study, only 45 (1.1%) had P-EGC. However, these tumors had distinct clinicopathologic features when compared with other types of EGC. Patients with P-EGC were older with higher body mass index (BMI) than those with non-P-EGC differentiated or undifferentiated tumors. The percentage of men in the P-EGC group (33/45 [73.3%]) was higher than undifferentiated EGC groups (1088/2113 [51.5%]). P-EGCs were located more commonly in the lower third of the stomach than other types of EGC (P-EGC: 32/45 [71.1%] vs differentiated EGC: 1185/2105 [56.3%] vs undifferentiated EGC: 774/2113 [36.6%], *P* < .001). Elevated gross morphology was especially prominent in P-EGC ((P-EGC: 21/45 [46.7%] vs differentiated EGC: 1185/2105 [56.3%] vs undifferentiated EGC: 774/2113 [49.6%], *P* < .001). Deeper submucosal invasion was significantly more common in P-EGC than in other types of EGC (sm1: 6/49 [13.3%]; sm2: 29/45 [64.4%], *P* < .001). Rates of angiolymphatic invasion and lymph node metastasis were higher in those with P-EGC than in those with other tumors, occurring in 21 (46.7%) and 9 (20.0%) patients, respectively (*P* < .001).

**Table 1:** Baseline clinicopathologic characteristics of subjects with papillary or non-papillary early gastric cancer.

Characteristic	Total (n = 4264)	Differentiated EGC (n = 2106, 49.4%)	Undifferentiated EGC (n = 2113, 49.6%)	Papillary EGC (n = 45, 1.1%)	*P* value
***Age (years***)					<.001
Mean ± SD	57.9 ± 11.7	61.7 ± 10.4	53.9 ± 11.7	64.5 ± 10.5	
***BMI (kg/m***^***2***^)					.003
Mean ± SD	24.0 ± 3.2	24.1 ± 3.1	23.9 ± 3.2	25.2 ± 3.8	
***Sex***					<.001
Male	2707 (63.5)	1586 (75.3)	1088 (51.5)	33 (73.3)	
Female	1557 (36.5)	520 (24.7)	1025 (48.5)	12 (26.7)	
***Location***					<.001
Upper	529 (12.4)	238 (11.3)	288 (13.6)	3 (6.7)	
Middle	1744 (40.9)	683 (32.4)	1051 (49.7)	10 (22.2)	
Lower	1991 (46.7)	1185 (56.3)	774 (36.6)	32 (71.1)	
***Size (cm***)					.415
Mean ± SD	3.3 ± 1.9	3.4 ± 1.9	3.3 ± 2.0	3.5 ± 1.8	
***Morphology***					<.001
Elevated	666 (15.6)	511(24.3)	134 (6.3)	21 (46.7)	
Flat	634 (14.9)	246 (11.7)	384 (18.2)	4 (8.9)	
Depressed	2964 (69.5)	1349 (64.1)	1595 (75.5)	20 (44.5)	
***Depth***					<.001
mucosa	2314 (54.2)	1027 (48.8)	1276 (60.4)	10 (22.2)	
sm1	485 (11.4)	299 (14.2)	180 (8.5)	6 (13.3)	
sm2	1466 (34.4)	780 (37.0)	657 (31.1)	29 (64.4)	
***Angiolymphatic invasion***					<.001
No	3613 (84.7)	1707 (81.1)	1882 (89.1)	24 (53.3)	
Yes	651 (15.3)	399 (18.9)	231 (10.9)	21 (46.7)	
***Lymph node metastasis***					<.001
No	3814 (89.4)	1912 (90.8)	1866 (88.3)	35 (80.0)	
Yes	450 (10.6)	194 (9.2)	247 (11.7)	9 (20.0)	

Values are indicated as n (%) unless otherwise indicated.

BMI = body mass index, EGC = early gastric cancer, SD = standard deviation, sm = submucosal invasion.

Risk factors associated with lymph node metastasis were identified using logistic regression analysis (Table 2). In the multivariate analysis, BMI ≥ 25 kg/m^2^ (OR: 1.3, 95% CI: 1.1–1.7), men (OR: 0.8, 95% CI: 0.6–1.0), middle stomach location (OR: 1.7, 95% CI: 1.1–2.5), lower stomach location (OR: 2.2, 95% CI: 1.5–3.3), flat gross morphology (OR: 0.7, 95% CI: 0.4–1.0), ALI (OR: 4.8, 95% CI: 3.7–6.1), and ulceration (OR: 1.7, 95% CI: 1.2–2.4) were associated with LNM. Other factors significantly associated with lymph node metastasis were submucosal invasion, including sm1 (OR: 2.1, 95% CI: 1.4–3.1), sm2 (OR: 3.9, 95% CI: 3.0–5.2), tumor size > 3 cm (OR: 2.0, 95% CI: 1.5–2.7), and undifferentiated EGC (OR: 2.1, 95% CI: 1.6–2.6). P-EGC was a marginally significant risk factor for lymph node metastasis (OR: 2.5, 95% CI: 1.0–6.5). Risk factors in univariate and multivariate logistic analyses were nearly the same.

**Table 2:** Analysis of clinicopathologic characteristics associated with lymph node metastasis.

Characteristic	Total	LNM	Univariate model		Multivariate model*	
	No.	No. (%)	OR	(95% CI)	OR	(95% CI)
***Age (years***)						
<65	2999	307 (10.2)	1			
≥65	1265	143 (11.3)	1.1	(0.9–1.4)		
***BMI (kg/m***^***2***^)						
<25	2748	261 (9.5)	1		1	
≥25	1516	189 (12.5)	1.4	(1.1–1.7)	1.3	(1.1–1.7)
***Sex***						
Female	1557	189 (12.1)	1		1	
Male	2707	261 (9.6)	0.8	(0.6–0.9)	0.8	(0.6–1.0)
***Location***						
Upper	529	39 (7.4)	1		1	
Middle	1744	166 (9.5)	1.3	(0.9–1.9)	1.7	(1.1–2.5)
Lower	1991	245 (12.3)	1.7	(1.2–2.5)	2.2	(1.5–3.3)
***Morphology***						
Elevated	666	91 (13.7)	1		1	
Flat	634	51 (8.0)	0.6	(0.4–0.8)	0.7	(0.4–1.0)
Depressed	2964	308 (10.4)	0.7	(0.6–0.9)	0.8	(0.6–1.1)
***Angiolymphatic invasion***						
No	3613	225 (6.2)	1		1	
Yes	651	227 (34.9)	8.1	(6.6–10.0)	4.8	(3.7–6.1)
***Ulcer***						
No	3997	390 (9.8)	1		1	
Yes	267	60 (22.5)	2.7	(2.0–3.6)	1.7	(1.2–2.4)
***Depth***						
Mucosa	2226	87 (3.8)	1		1	
sm1	434	51 (10.5)	3.0	(2.1–4.3)	2.1	(1.4–3.1)
sm2	1154	312 (21.3)	6.9	(5.4–8.9)	3.9	(3.0–5.2)
***Tumor diameter (cm***)						
≤ 2	1286	75 (5.8)	1		1	
2.1–3	1019	104 (10.2)	1.8	(1.3–2.5)	1.3	(1.0–1.9)
>3	1959	271 (13.8)	2.6	(2.0–3.4)	2.0	(1.5–2.7)
***Histologic type***						
Differentiated	2106	194 (9.2)	1		1	
Undifferentiated	2113	247 (11.7)	1.3	(1.1–1.6)	2.1	(1.6–2.6)
Papillary	27	8 (29.6)	4.2	(1.8–9.6)	2.5	(1.0–6.3)
Papillary mixed with differentiated type	10	1 (11.1)	1.0	(0.1–11.0)	1.0	(0.1–10.0)
Papillary mixed with undifferentiated type	8	0 (0.0)	-	-	-	-

* Adjusted for BMI, sex, location, morphology, angiolymphatic invasion, ulcer, depth, size, and histology.

BMI = body mass index, CI = confidence interval, LNM = lymph node metastasis; No., number, OR = odds ratio.

We also analyzed lymph node metastasis or angiolymphatic invasion according to the ESD criteria (Table 3). Overall, 1482 patients met the indication criteria for ESD (absolute or expanded), including 13 who had P-EGC and 1469 who had non-P-EGC. Lymph node metastasis and angiolymphatic invasion rates of P-EGC were not significantly different from those of non-P-EGC (*P = *.2 and *P* = .07, respectively). When analyzed separately according to whether absolute or expanded ESD indication criteria were met, P-EGC meeting expanded criteria had higher lymph node metastasis rate (1/10, 10%) and angiolymphatic invasion rate (2/10, 20%) than non-P-EGC meeting absolute or expanded ESD criteria (*P = *.03 and *P* *< *.001, respectively).

**Table 3:** Association between lymph node metastasis or angiolymphatic invasion and tumor type according to endoscopic submucosal dissection indication criteria.

Tumor type and criteria status	LNM		ALI	
	Number (%)	*P* value	Number (%)	*P* value
***Tumors meeting ESD indication criteria (total***)*				
Non-P-EGC (n = 1469)	29 (2.0)	0.2	62 (4.2)	0.07
P-EGC (n = 13)	1 (7.7)		2 (15.4)	
***Tumors meeting ESD indication criteria according to criteria category*** †				
Non-P-EGC (absolute) (n = 366)	1 (0.3)	0.03	7 (1.9)	<.001
Non-P-EGC differentiated (expanded) (n = 785)	20 (2.5)		53 (6.8)	
Undifferentiated EGC (expanded) (n = 318)	8 (2.5)		2 (0.6)	
P-EGC (absolute) (n = 3)	0 (0)		0 (0)	
P-EGC (expanded) (n = 10)	1 (10.0)		2 (20.0)	

ALI = angiolymphatic invasion, EGC = early gastric cancer, ESD = endoscopic submucosal dissection, LNM = lymph node metastasis, P-EGC = papillary early gastric cancer.

* Tumors meeting absolute criteria plus tumors meeting expanded criteria.

† Tumors meeting the indication criteria for ESD, classified by indication category (absolute or expanded).

Of the 9 P-EGC patients with lymph node metastasis, all had a submucosal invasion. Lymph node metastasis was present in 2 (33.3%) of 6 patients with sm1 (Table 4). Tumors in these 2 patients were 2 cm and 4 cm in diameter. Ulcers were present in none of these P-EGCs with lymph node metastasis and submucosal invasion.

**Table 4:** Lymph node metastasis of papillary early gastric cancers according to depth of tumor invasion.

n (%)	Mucosa (n = 10)	sm1 (n = 6)	sm2 (n = 29)	*P* value
LNM	0 (0)	2 (33.3)	7 (24.1)	.890
Ulcer	1 (10)	1 (25.0)	4 (13.7)	.176
Ulcer without LNM	1	1	4	
Ulcer with LNM	0	0	0	

LNM = lymph node metastasis, sm = submucosal invasion.

## 4. Discussion

In our study, P-EGC was associated with higher rates of both lymph node metastasis and submucosal invasion than other types of EGC. In the multivariate analysis, non-mixed-type P-EGC was a marginally significant risk factor for lymph node metastasis. Lymph node metastasis also differed significantly according to BMI and tumor location. When the safety of ESD was examined, P-EGC meeting expanded indication criteria was found to have higher rates of lymph node metastasis and angiolymphatic invasion than non-P-EGC meeting ESD criteria. None of the P-EGCs with lymph node metastasis had ulceration. There was no apparent relationship between lymph node metastasis and the depth of submucosal invasion. Therefore, ESD might not be suitable for treating P-EGC that meets expanded indication criteria.

Several studies have investigated the use of ESD for P-EGC.^[11,16–18]^ Clinicopathologic characteristics of P-EGC reported in other studies were similar to those observed in our study. It has been reported that P-EGC is associated with higher rates of lymphatic invasion and submucosal invasion than undifferentiated or other differentiated EGCs.^[8,10,11,17]^ Several prior studies have noted that angiolymphatic invasion is a significant risk factor for lymph node metastasis in P-EGC.^[9–12,17,19]^ A 2019 meta-analysis found an increased risk of submucosal invasion and lymph node metastasis in P-EGC than in tubular EGC, especially in Asians.^[8]^ Furthermore, curative resection rates with ESD were lower for P-EGC (49.6%–69.5%) than for other EGCs.^[11,16]^

Nevertheless, the risk of lymph node metastasis in P-EGC varied in previous studies. One study reported no increased risk of lymph node metastasis compared with other histologic types.^[17]^ However, that study did not report any lymph node metastasis in P-EGC with sm1 invasion or how lymph node dissection proceeded during surgery.^[17]^ Other studies reported that P-EGC was associated with an increased risk of lymph node metastasis than undifferentiated or other differentiated EGCs (similar to our findings).^[8,11]^ One study found that 2 of 6 P-EGCs with sm1 invasion had lymph node metastasis or lymphatic invasion,^[11]^ similar to our findings. Among P-EGCs that correspond to ESD criteria in 2 studies,^[11,17]^ lymph node metastasis was found only in P-EGCs that met the expanded criteria. A recent multicenter retrospective study reported that submucosal P-EGC had higher rate of lymph node metastasis and different features than other differentiated submucosal EGCs.^[19]^ Another study reported that 30% of patients with P-EGC meeting expanded ESD indication criteria had lymphatic invasion or lymph node metastasis.^[11]^ In contrast, a recent article suggested that ESD for P-EGC using the Japanese guidelines produced favorable long-term outcomes, with no extra-gastric lymph node metastasis and a lower rate of metachronous recurrence (5.2%) during a median follow-up of 58 months.^[16]^ Therefore, the safety of ESD remains controversial. In view of other risk factors for lymph node metastasis, some studies have reported that women are at risk for lymph node metastasis,^[20]^ similar to our results. This is due to estrogen’s effect on proliferation and invasion of gastric cancer, which could be prevented by administration of tamoxifen, an estrogen receptor antagonist.^[21]^ A high BMI was also a risk factor for lymph node metastasis in our study. This might be explained by hormonal and biological processes related to tumor cell proliferation such as breast cancer.^[22]^ The depth of tumor invasion and the diameter of cancer are well-known risk factors for lymph node metastasis of EGC.^[23]^ In addition, previous studies have demonstrated that macroscopic type and differentiation are independent risk factors affecting lymph node metastasis.^[24]^ Tumor at body or lower third region has a tendency of more lymph node metastasis than that at the other locations,^[25]^ consistent with our results.

P-EGCs in our study showed aggressive features, including significantly higher rates of angiolymphatic invasion and lymph node metastasis, consistent with findings of other studies.^[11,12]^ However, why lymph node metastasis occurs with P-EGC has not been clarified yet. Several molecular studies have been conducted to evaluate the behavior of papillary gastric cancer. Higher and more widespread high-frequency microsatellite instability might be associated with clinical behavior of papillary gastric cancers.^[26]^ A high incidence of BRM deficiency with an antineoplastic role might contribute to an aggressive behavior through epigenetic regulations.^[27]^ Phagocytosis-mediated chromosomal instability and aneuploidy could cause the aggressive behavior of micropapillary gastric carcinoma.^[28]^ Further studies are needed clarify the pathogenesis of papillary gastric cancer.

When limited to patients with EGC meeting criteria for ESD (absolute or expanded), the rate of lymph node metastasis was approximately 2.8-fold higher for P-EGC than for other types of EGCs. However, P-EGC meeting expanded indication criteria for ESD was associated with a 29-fold higher likelihood of lymph node metastasis than non-P-EGC meeting absolute indication criteria (data not shown). In our study, P-EGC with lymph node metastasis that met the current ESD criteria had a tumor size of 2 cm and sm1 invasion. In previous studies, there were less than 2 cases of P-EGC with lymph node metastasis that met curative ESD criteria and the tumor size was >3 cm or with sm1 invasion.^[11,17]^ These findings suggest that it could be dangerous to apply expanded ESD criteria for P-EGCs. Indeed, lymph node metastasis was observed in 2 of 6 patients with sm1 P-EGCs in our study. Additionally, no ulcer was observed in any of our P-EGC patients with lymph node metastasis or submucosal invasion, consistent with findings of previous studies reporting that ulcers were less common in P-EGC and that ulcers were not significant risk factors for lymph node metastasis in P-EGC, in contrast with their frequency and role in other EGCs.^[11,17]^ Therefore, the current expanded ESD guidelines do not seem to be appropriate for P-EGCs as they could not determine the correct depth of the tumor prior to ESD. Although a recent study reported fewer recurrences following ESD for P-EGC, the study included only 15 tumors that met the expanded indication criteria.^[16]^ Five-year survival rates after surgical resection were significantly worse for P-EGC than for tubular EGC.^[9]^ Thus, there is a clear need for long-term follow-up studies to guide the establishment of appropriate indication criteria for ESD as a treatment option for P-EGC.

This study has several limitations, including its retrospective, single-center design. Furthermore, the number of P-EGCs was relatively small compared to other histologic types. Moreover, we investigated only gastrectomy specimens, not ESD specimens. In addition, long-term outcomes such as recurrence and survival following ESD were not assessed. Owing to the exclusion of ESD specimens, differentiated non-P-EGC tended to have higher lymph node metastasis than undifferentiated EGC. However, a large-scale evaluation of consecutive patients with EGC was conducted and their postoperative pathologic data were analyzed. The limitation was having only 1 case of P-EGC with lymph node metastasis in the expanded ESD criteria. However, previous studies have shown few P-EGCs with lymph node metastasis meeting the curative ESD criteria because P-EGC is a rare histologic type among gastric cancer. Therefore, expanded indication criteria for ESD that are currently accepted do not appear to be appropriate for treating P-EGC examined in our study.

## 5. Conclusion

In summary, ESD should be considered carefully for P-EGC treatment because of the high rate of submucosal invasion and the likelihood of lymph node metastasis associated with these tumors, especially in those falling within expanded indication criteria for ESD. Our results suggest that ESD might be a suitable therapeutic option only when P-EGC meets the absolute indication criteria for ESD. We recommend surgery for patients with P-EGC who meet expanded ESD indications.

## Author contributions

All authors performed the research. JH Lee performed manuscript writing and data analysis. JY Oh performed data analysis. YI Kim, JY Lee, CG Kim, IJ Choi, JW Ryu, and Y-W Kim performed data analysis and critical revision. S-J Cho was involved in writing of the manuscript, drafting conception and design, and obtaining funding.

**Conceptualization:** Soo-Jeong Cho.

**Data curation:** Ju Yeon Oh.

**Formal analysis:** Jung Hwan Lee, Young-Il Kim, Jong Yeul Lee, Chan Gyoo Kim, Il Ju Choi, Keun Won Ryu, Young-Woo Kim.

**Funding acquisition:** Soo-Jeong Cho.

**Methodology:** Soo-Jeong Cho.

**Writing – original draft:** Jung Hwan Lee, Soo-Jeong Cho.

**Writing – review & editing:** Young-Il Kim, Jong Yeul Lee, Chan Gyoo Kim, Il Ju Choi, Keun Won Ryu, Young-Woo Kim, Soo-Jeong Cho.
